# Synthetic Cannabinoid Abuse and a Rare Alpha-1-Antitrypsin Mutant Causing Acute Fulminant Hepatitis: A Case Report and Review of the Literature

**DOI:** 10.1155/2017/9627452

**Published:** 2017-12-03

**Authors:** Kurt J. Knowles, Eric X. Wei, Abhishek Seth, John Bienvenu, James Morris, Kenneth Manas, Paul Jordan, Moheb Boktor

**Affiliations:** ^1^Department of Pathology, Louisiana State University Health Science Center, Shreveport, LA 71103, USA; ^2^Department of Gastroenterology and Hepatology, Louisiana State University Health Science Center, Shreveport, LA 71103, USA

## Abstract

Synthetic cannabinoids (SCs) abuse is on the rise because they are easily obtained over the counter; they are potent psychoactive compounds and routine drug testing does not detect them. As their abuse is on the rise, so are their detrimental side effects; however, the occurrence of acute hepatitis due to SCs abuse has been reported only once before. In this case, testing revealed that the patient was also heterozygous for alpha-1-antitrypsin (A-1-AT) with the phenotype of PI⁎EM. This mutant phenotype has never been reported as a cause of A-1-AT disease and the abuse of SCs in a patient with this phenotype has also never been reported. This case illustrates the possible need to expand routine drug testing for SCs and consider A-1-AT phenotyping in certain clinical scenarios.

## 1. Introduction

SCs are also known as spice or K2, and their abuse has increased over the past few decades; however, they are associated with severe toxicity including seizures, cardiac arrhythmias, acute kidney injury, extreme agitation, and shortness of breath [1], but acute liver failure has only been reported once before [2]. The association with mutant phenotypes of alpha-1-antitrypsin has never been reported before and the phenotype Pi*∗*EM has never been associated with disease of any type. This case report describes the clinical and pathologic consequences of using SCs in a patient with Pi*∗*EM phenotype.

## 2. Case Report

**Figure 1:**
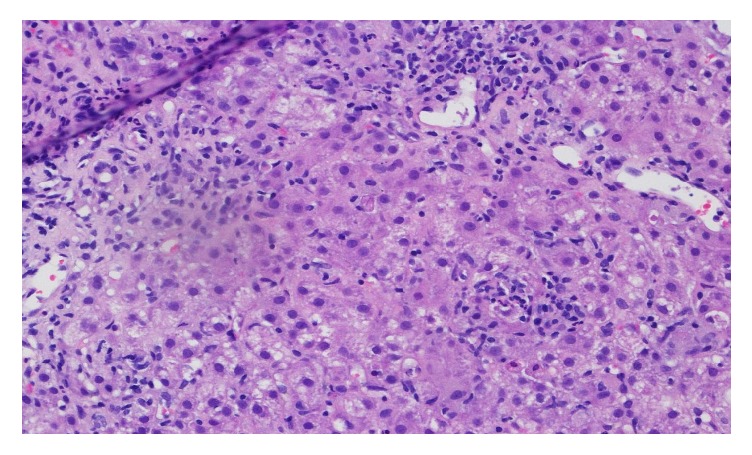
Liver biopsy. Severe inflammatory response comprised of neutrophils and lymphocytes with hepatocellular necrosis. No plasma cells or eosinophils and no bile duct damage or fibrosis. H&E stain, ×400.

**Figure 2:**
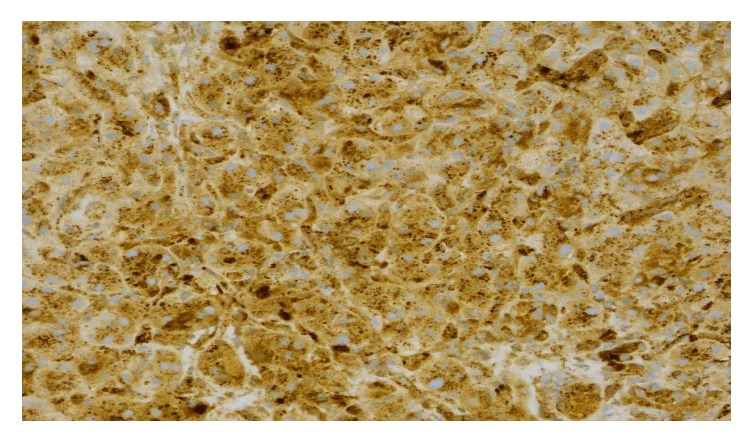
Immunohistochemistry for alpha-1-antitrypsin proteins. Diffuse strong positivity within hepatocytes and Kupffer cells indicated by the brown staining. IHC, ×400.

A patient with a history of synthetic marijuana abuse was admitted for hepatitis and jaundice. On admission, the total bilirubin was 20.4, ALT was 1922, and AST was 1631 (see Table 1 for additional laboratory findings). CT scan, gallbladder ultrasound, and MRCP did not reveal any abnormal findings. Extensive viral workup including anti-IgM antibodies for CMV, EBV, varicella, and HSV1 and HSV2 was all negative. Antigens for HIV, HCV RNA, and HBV DNA were negative. The serum ceruloplasmin was normal, ruling out Wilson's disease; hereditary hemochromatosis DNA mutation was absent; ANA titers were negative; and antismooth muscle antibodies were weakly positive. The serum anti-1-antitrypsin was low-normal. A liver biopsy was performed which demonstrated severe acute and chronic hepatitis, destruction of the limiting plate, and hepatocyte necrosis, but initially without a specific etiology (Figure 1). There was no significant plasmacytic infiltrate, no eosinophils, no bile duct destruction, and no segmental fibrosis of the bile ducts. The lack of these features ruled out autoimmune hepatitis, primary biliary sclerosis, and primary sclerosing cholangitis. The PAS stain with and without diastase demonstrated intracytoplasmic granules, which is nonspecific but raised the possibility of alpha-1-antitrypsin deficiency. In order to confirm whether the PAS-positive granules were alpha-1-antitrypsin proteins, immunohistochemistry for alpha-1-antitrypsin was performed, which was definitely positive (Figure 2). Phenotyping for alpha-1-antitrypsin was performed and the patient was heterozygous PiEM (normal PiMM). The patient was treated with intravenous N-acetylcysteine with slow resolution of liver function, recovered, and was discharged 10 days later.

## 3. Discussion

SCs, also known as spice or K2, are a diverse group of chemicals that act on the central nervous system's cannabinoid receptors but are significantly more potent than naturally occurring delta-9-tetrahydrocannabinol. Their abuse has increased over the past few decades in part because in some states they can be obtained over the counter and routine drug testing does not detect them. Toxicity includes seizures, cardiac arrhythmias, acute kidney injury, extreme agitation, and shortness of breath [1], but acute liver failure has only been reported once before [2].

A-1-AT deficiency is very rare and occurs in about 1 of every 5–10,000 people. There are over one hundred A-1-AT phenotypes with 90% of normal individuals PiMM, and the PiEM phenotype has never been reported to cause disease. The PiZZ phenotype produces only 20% of the normal alpha-1-antitrypsin serum protein levels and this phenotype is responsible for causing chronic liver disease in 95% of the cases. The cause of liver disease in these patients is the accumulation of abnormal proteins in the cytoplasm of hepatocytes with the activation of several cellular processes that attempt degradation of the abnormal proteins [3]. The cellular processes involved are the autophagy response, inflammatory response, unfolded protein response (UPR), and the role of chaperones in protein quality control.

Several studies have demonstrated that the mitochondria and endoplasmic reticulum are intimately associated structurally and biochemically through the mitochondria-ER associated membrane or MAM [4–6]. The MAM is responsible for calcium and ion exchanges between the ER and the mitochondria, which regulates proper protein folding and secretion, mutant protein degradation, and apoptosis. Oxidative stress disrupts the calcium and ion equilibrium which has numerous effects on the ER. SCs undergo mitochondrial P450 mediated oxidative metabolism predominantly by the human liver microsomes CYP2C9 and CYP1A2 and minimally by CYP2C19 [7]. These drugs and their metabolites create an oxidative stress on the function of mitochondria, the ER, and the MAM, which is resolved when the patient ceases using SCs and starts treatment with N-acetylcysteine which restores the oxidative capacity of the liver and normalizes the function of the ER and MAM as demonstrated by the successful use of N-acetylcysteine by our institution and also as reported by Sheikh et al. [2].

This oxidative stress has several cellular effects that impact the metabolism of A-1-AT and the mutant protein forms. One of the effects on the ER involves the function of chaperones. Chaperones are responsible for protein quality control. Wild-type proteins are folded properly and then secreted into the cytosol whereas mutant proteins are processed for degradation or autophagy. These functions are controlled by the MAM and when disrupted do not occur effectively. Schmidt demonstrated that mutant A-1-AT ZZ forms interact with the Grp78, Grp94, and Grp170 chaperones, calnexin, and UGGT [8]. Thus, oxidative stress interferes with proteins responsible for the proper metabolism of mutant A-1-AT which may then accumulate in the hepatic cytosol. Another effect of stress is the activation of the unfolded protein response or UPR. As demonstrated by Burton et al. [5], activation of the UPR has several effects: it increases proinflammatory cytokines against the cells, increases reactive oxygen species which decreases ATP production which in turn inhibits antioxidative responses, and leads to stimulation of chaperone production in order to metabolize mutant proteins [8]. However, continued oxidative stress may also result in the accumulation of unfolded proteins and lead to apoptotic stimuli [9]. Thus, by interfering with chaperone function and activation of the UPR, oxidative stress causes mutant A-1-AT retention in hepatocytes, increases the inflammatory response and autophagy, and decreases the energy production needed to combat the stress. If the stress is prolonged, severe hepatocellular necrosis will ensue and continue until the cause of the oxidative stress is removed or ameliorated.

## 4. Conclusions

There are several clinical implications and/or questions raised by this case. First, all patients who are suspected to suffer from an adverse drug reaction but their urine and serum drug screening is negative need to be tested for SCs, and laboratories need to develop methodologies to do so. As the use of SCs will continue to increase, clinicians need all available tools to provide optimal care for their patients. Second, if the serum A-1-AT protein level is below normal or borderline normal, A-1-AT phenotyping should be considered if the degree of hepatotoxicity seems to be in excess of the known insult. Finally, if increasing oxidative stress on ER, MAM, and mitochondria increases the precipitation of mutant A-1-AT forms, can reduction of oxidative stress and induction of the cytochrome p450 system decrease the accumulation of mutant A-1-AT forms and ameliorate hepatotoxicity in patients homozygous for Pi*∗*ZZ?

## Figures and Tables

**Table 1:** 

	Patient result	Normal range
Total bilirubin	20.4	0.2–1.0
Direct bilirubin	15.1	0.0–0.3
Indirect bilirubin	5.3	1.0–0.7
ProTime	15	10.0–13.2
INR	1.29	0.9–1.2
AST	1031	12–37
ALT	1922	12–78
Alk. phos.	123	12–176
ANA	Negative	Negative
ASMH	27	Negative, 0–19
		Weakly positive, 20–30
		Strongly positive, >30
Ceruloplasmin	23.2	16.0–31.0
Hereditary hemochromatosis mutation	Negative	Negative
Acetaminophen	<2	10–30

## Additional Points

*Lay Summary*. Synthetic marijuana use has only been reported to cause acute liver failure once.
